# A Janus-Type Phthalocyanine for the Assembly of Photoactive DNA Origami Coatings

**DOI:** 10.1021/acs.bioconjchem.1c00176

**Published:** 2021-05-24

**Authors:** Asma Rahali, Ahmed Shaukat, Verónica Almeida-Marrero, Bassem Jamoussi, Andrés de la Escosura, Tomás Torres, Mauri A. Kostiainen, Eduardo Anaya-Plaza

**Affiliations:** ∥ Department of Organic Chemistry, Universidad Autónoma de Madrid (UAM), Calle Francisco Tomás y Valiente 7, 28049 Madrid, Spain; ‡ Didactic Research Laboratory of Experimental Sciences and Supramolecular Chemistry (UR17ES01), University of Carthage, Faculty of Sciences Bizerte, Zarzouna, 7021 Bizerte, Tunis; ∫ Department of Bioproducts and Biosystems, Aalto University, Kemistintie 1, 02150 Espoo, Finland; ⊥ Department of Environmental Sciences, Faculty of Meteorology, Environment and Arid Land Agriculture, King Abdulaziz University, 21589 Jeddah, Saudi Arabia; § Institute for Advanced Research in Chemical Sciences (IAdChem). Universidad Autónoma de Madrid (UAM), Campus de Cantoblanco, 28049 Madrid, Spain; ∓ IMDEA-Nanociencia, Campus de Cantoblanco, 28049 Madrid, Spain

## Abstract

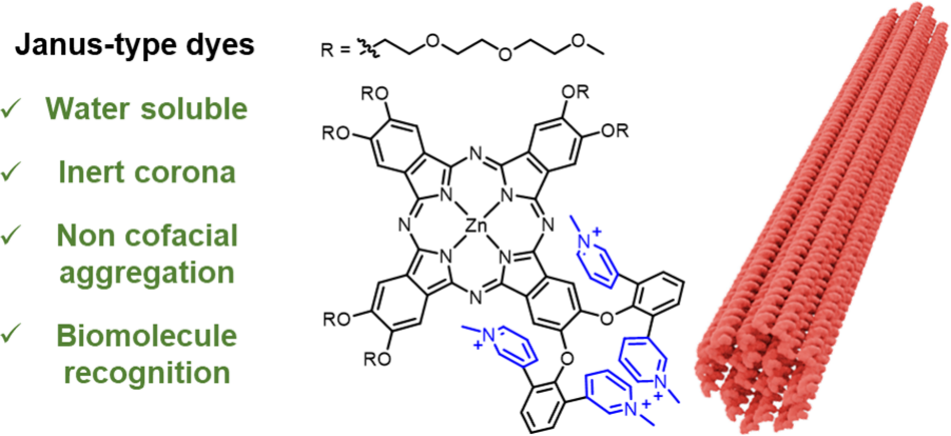

Design and synthesis of novel photosensitizer architectures is a key step toward new multifunctional molecular materials. Photoactive Janus-type molecules provide interesting building blocks for such systems by presenting two well-defined chemical functionalities that can be utilized orthogonally. Herein a multifunctional phthalocyanine is reported, bearing a bulky and positively charged moiety that hinders their aggregation while providing the ability to adhere on DNA origami nanostructures via reversible electrostatic interactions. On the other hand, triethylene glycol moieties render a water-soluble and chemically inert corona that can stabilize the structures. This approach provides insight into the molecular design and synthesis of Janus-type sensitizers that can be combined with biomolecules, rendering optically active biohybrids.

## Introduction

Phthalocyanines (Pc) are porphyrinoid derivatives, composed of four isoindole units connected through nitrogen atoms, and an inner coordination site able to accommodate a wide variety of metal atoms.^1,2^ The resulting extended aromatic surface is responsible for their high extinction coefficient in the red/near-infrared region of the UV–vis spectrum, which, together with their rich electrochemistry and stability, makes them outstanding candidates for a variety of applications in optoelectronics,^3−5^ catalysis,^6−8^ sensing,^2^ and biomedicine.^9−15^ In the latter, conjugation of Pcs and diverse biomolecules enhance desired properties such as biocompatibility, biodistribution, and targeting of photogenerated reactive oxygen species such as singlet oxygen (^1^O_2_).^16^ Among the vast array of biomolecules available, nucleic acid derivatives and, in particular, DNA origami has been at the center of extensive research in recent years.^17−21^ DNA origami preparation is based on the folding of a long, circular single stranded DNA (“scaffold DNA”) by customized short (15–60 nucleotides) single stranded oligonucleotides (“staple strands”).^22−24^ This technology yields almost any well-defined arbitrary nanoscale shape in highly parallel fashion. Due to its multimodal nature and nanometre-scale accuracy, DNA origami nanostructures are directly relevant for drug delivery,^25,26^ imaging,^21,27^ biophysical studies,^28,29^ material science,^30^ and nanostandards.^31^

Hybrids of Pc with DNA origami have been scarcely investigated despite the evident synergy between the moieties. We have previously reported that symmetric octacationic zinc(II) Pcs (ZnPc) and DNA origami structures can be assembled through electrostatic interactions, yielding enhanced optical properties and origami protection against enzymatic digestion.^32^ Thus, the development of Pc derivatives with better architectural and functional control of their optical properties is a desirable goal. Herein we describe the design and synthesis of a Janus-type Pc with sharp differences in the chemical nature of the functional groups placed at opposite sides of the molecule. Furthermore, we show that the Pcs are able to bind electrostatically on the surface of DNA origami structures, which modulates their optical properties.

## Results and Discussion

### Design of the Janus-Type Moiety and DNA Origami

**Figure 1:**
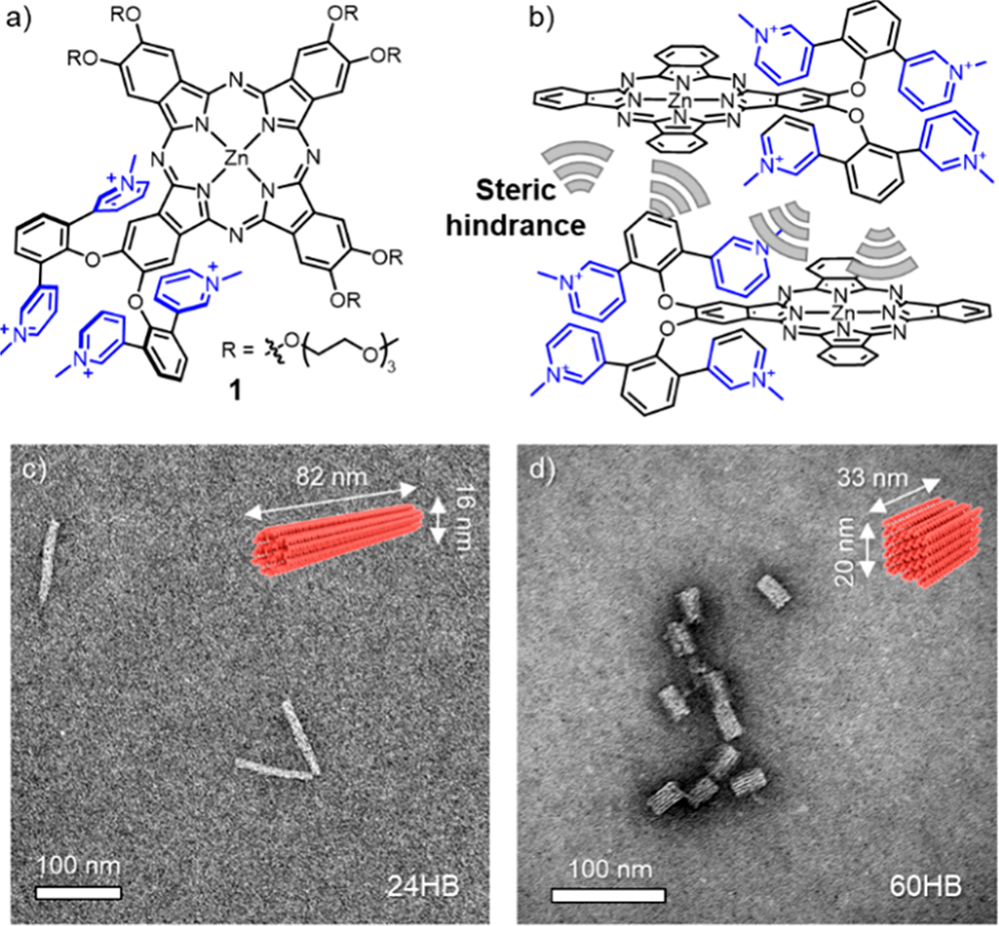
(a) Chemical structure of **1**. (b) Schematic representation of the bulky cationic groups hindering the cofacial aggregation. Triethylene glycol monomethyl ether chains and iodide counterions are omitted for clarity. Structures and dimensions of (c) 24HB and (d) 60HB.

The chemical design of novel Pcs results in derivatives with enhanced performance for targeted applications. For instance, axial or peripheral modification with hydrophilic groups renders aqueous solubility of the otherwise hydrophobic macrocycles.^33,34^ More refined architectures can provide additional control on the interaction with the environment, either by tuning their self-assembly or providing recognition/targeting moieties. In this study, a Janus-type ZnPc (**1**) was designed, synthesized, and characterized. The synthesized molecule displays a Janus-type architecture, presenting different functional groups on opposite sides of the molecule: on one side, a bulky and positively charged region, located on a single isoindole; on the other side, a total of six triethylene glycol chains on the remaining three isoindole moieties (Figure 1a), rendering a water-soluble, inert, and biocompatible surface. The charged substitution pattern indeed provides a rigid and bulky frame that hinders the typical aggregation of these derivatives in aqueous media (Figure 1b). This ensures that the outstanding photophysical properties of Pcs are maintained and not quenched upon self-assembly.^35^ Additionally, the four densely packed charges are suitable to undergo electrostatic recognition with negatively charged DNA origami structures. Here we have studied two model structures: 24- and 60-helix bundles (24HB and 60HB, respectively). The 24HB was obtained by one-pot thermal annealing, folding a long scaffold of 7560 nucleotides with the help of 202 oligonucleotide staple strands.^36^ This yields a rigid cylindrical nanostructure of 82 nm length and 16 nm diameter (Figure 1c). The 60HB synthesis was achieved adapting previously reported methodology,^37^ using a 7249 nucleotide scaffold and 141 staple strands. This results in a brick-like structure with 20 nm height and 33 nm length (Figure 1d). For further details, refer to SI.

### Synthesis of the Janus-Type Molecule

**Scheme 1:**
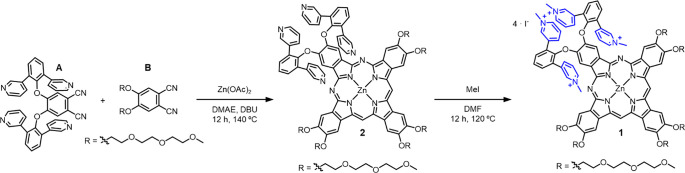
Synthesis of ZnPc **1** Asymmetric ZnPc **2** was obtained after separation from statistical cyclotetramerization of **A** and **B** in the presence of Zn(OAc)_2_. Cationic ZnPc **1** was obtained after quaternization of the pyridinyl moieties in the presence of MeI.

The synthesis of **1** (Scheme 1) was achieved by statistical cyclotetramerization of phthalonitrile **A**, containing four pyridyl groups, and **B** six (neutral and biocompatible) triethylene glycol (TEG) monomethyl ether chains. Briefly, **A** was prepared by microwave-assisted nucleophilic aromatic substitution of 4,5-dichlorophthalonitrile with 2,6-bis(3-pyridinyl)phenol.^38^**B** was prepared by Rosenmund–von Braun cyanidation of the dibromo aromatic derivative (see SI for details). The statistical cyclotetramerization of **A** and **B** was carried out at 140 °C for 12 h, in 2-dimethylaminoethanol (DMAE) with 1,8-diazabicyclo [5.4.0]undec-7-ene (DBU) as a base. The resulting AB_3_ derivative (**2**) was obtained in 15% yield, after separation from the other ZnPc compounds formed (mostly, the B_4_ and the ABAB/A_2_B_2_ derivatives) through column chromatography in silica gel. A mixture of chloroform/methanol/pyridine (100:5:1) was employed as eluent. Subsequent purification was achieved by size exclusion chromatography performed in Biobeads with chloroform as eluent. Last, methylation of the pyridyl groups was performed with methyl iodide in dry DMF, yielding **1** in 87% yield. Compound **1** was characterized by means of ^1^H NMR in DMSO-*d*_6_, high-resolution mass spectrometry (HR-MS), and infrared (IR) spectroscopy (see SI).

### Optical Characterization

**Figure 2:**
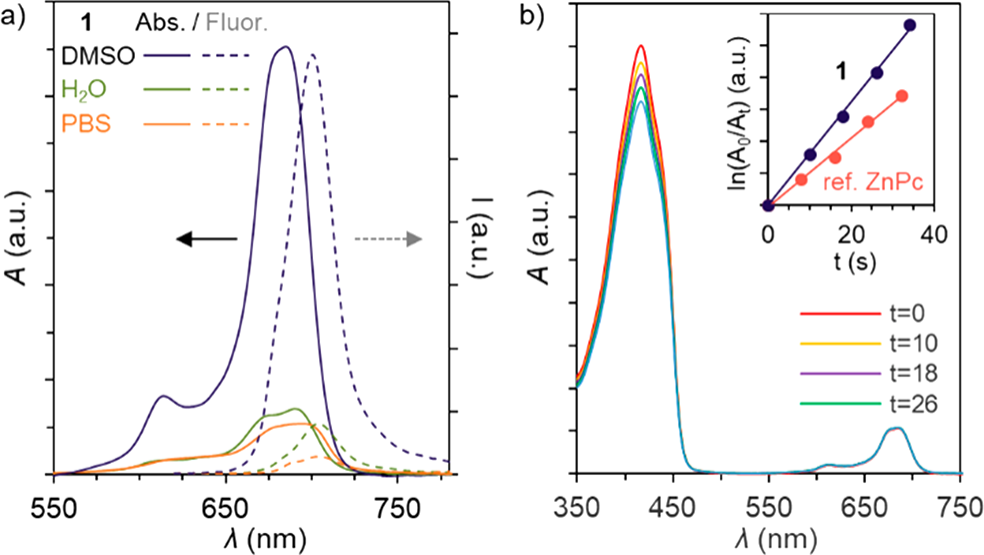
(a) UV–vis (solid) and emission (dotted) spectra of the Janus-type ZnPc **1** in DMSO (black), water (blue) and PBS (red). [**1**] = 3.6 (UV–vis) and 1.8 μM (emission). λ_exc_ = 655 nm. (b) DPBF absorption decrease at 414 nm, photoinduced by **1** in DMSO. Inset: plot of the DPBF absorption decay induced by **1** (black) and reference ZnPc (red).

To study the aggregation of **1**, the UV–vis absorption spectrum in DMSO, water, and 1x phosphate-buffered saline (PBS) was recorded (see Figure 2a). In DMSO, the spectrum showed an intense Q-band at 685 nm, characteristic of nonaggregated monomeric ZnPc. In aqueous medium, on the other hand, **1** presented a split Q-band, as consequence of the asymmetric substitution pattern of the macrocycle. Additionally, it shows a diminished absorption coefficient (ε) and broader features of the Q-band than in pure DMSO, with the absorbance maximum shifting to 690 and 694 nm in water and PBS, respectively. These spectral features are indicative of molecular crowding due to the hydrophobic nature of Pc, occurring to a different extent depending on the conditions. However, no bathochromic shift to ca. 630 nm of the absorption maximum was observed, as traditionally shown in cofacial ZnPc aggregation.^39^ The same behavior was observed in water with increasing concentrations of NaCl (Figure S5).

The fluorescence spectra of **1** was recorded in the same three solutions (Figure 2a). In DMSO, **1** showed an emission centered at 700 nm upon excitation at 665 nm. A fluorescence quantum yield (φ_F_) value of φ_F_ = 0.09 was calculated by adapting the method of Williams (see Experimental Section), irradiating the sample at 665 nm and with nonsubstituted ZnPc as reference (φ_F_ = 0.18, Figure S1). In aqueous media, both absorption and fluorescence decrease drastically. In water (Figure 2a, blue), the absorbance/emission ratio is maintained when compared to DMSO. This is a consequence of the aggregation hindrance of the cationic groups, avoiding the fluorescence quenching by exciton coupling. The same spectra in 1x PBS buffer (Figure 2a, orange) show a clear decrease in the fluorescence while revealing small changes in the absorption spectrum. This effect can be explained by agglomeration or crowding of **1** caused by the higher ionic strength of 1x PBS. A similar effect was observed with pyrene-substituted cationic ZnPcs.^40^ The molecules are close enough to undergo exciton coupling, while the chemical design hinders cofacial aggregation or H-type stacking.

### Photoactivity

Finally, the photoinduced ^1^O_2_ quantum yield (φ_Δ_) of **1** was determined in DMSO, using the relative method (see SI for details). The photodegradation of a molecular scavenger such as 1,3-diphenylisobenzofuran (DPBF) was recorded as a function of the irradiation time (Figure 2b). It shows a ^1^O_2_-induced bleaching at 414 nm (Figure 2b inset), while absorption of **1** remains intact, indicating no relevant photobleaching of the dye. ZnPc **1** shows a φ_Δ_ of 0.44, calculated with respect to the nonsubstituted ZnPc as reference (φ_Δ_(DMSO) = 0.67). This value highlights the efficient performance of **1** as photosensitizer, despite the bulky peripheral decoration.

### Biohybrid Characterization

**Figure 3:**
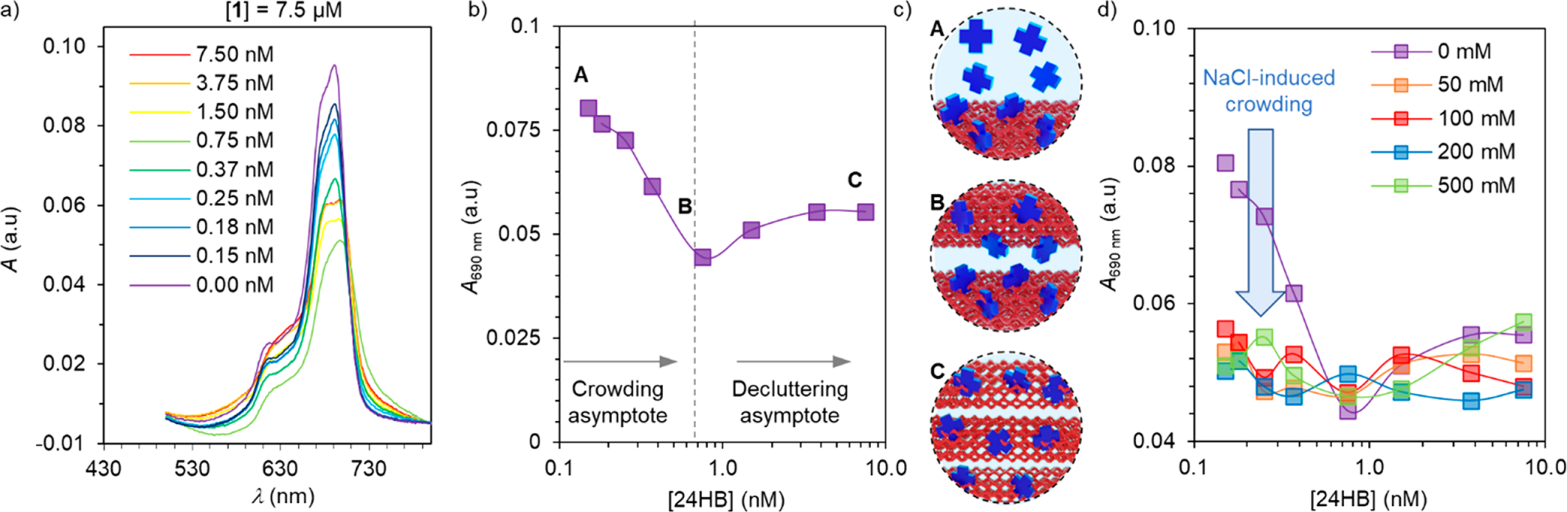
(a) Absorption spectra of 7.5 μM **1** in the presence of 0–7.50 nM of 24HB. (b) Absorbance of 7.5 μM of **1** at 690 nm in the presence of different 24HB concentrations. (c) Top to bottom: schematic representation of the **1**–24HB hybrids at (A) 40 000, (B) 10 000 and (C) 2000 equiv of **1** per 24HB. (d) Absorbance of 7.5 μM of **1** at increasing amounts of 24HB and NaCl. The solvent used for all experiment is aqueous 10% DMSO.

The interaction between **1** and DNA origami was studied by UV–vis spectroscopy in DMSO/water (1:9). In particular, the absorption of **1** at a constant concentration of 7.5 μM was recorded in the presence of increasing concentrations of 24HB (Figure 3a) and 60HB (Figure S7a). The ZnPc showed no significant band shift. However, by plotting the absorbance at 690 nm against the 24HB concentration (Figure 3b), two clear trends were observed (similar results were obtained for 60HB). First, the addition of 24HB up to 1.5 nM (10 000 equiv) results in a decrease in the apparent extinction coefficient (ε). This behavior, named *binding asymptote*, is the consequence of an excess of **1** binding stepwise to the DNA origami (Figure 3c, top). The saturation point (Figure 3c, middle) represents the complex with densely packed ZnPc, showing an apparent minimum ε. A further increase of the 24HB concentration (up to 7.5 nM, 50 000 equiv) resulted in an apparent ε increase (*decluttering asymptote*). In this regime, the available DNA origami surface area increases, diminishing the molecular packing of **1** (Figure 3c, bottom). This was further studied by repeating the above-mentioned experiments in the presence of increasing amounts of NaCl (Figure 3d and Figures S6 and S7). The binding asymptote was practically nonexistent above 50 mM of NaCl, because of the salt-induced aggregation of free **1** (Figure 3d). On the other hand, the decluttering asymptote was maintained, pointing toward a clear binding resilience when the ionic strength of the medium is high. This can be explained by the formation of a stable, kinetically trapped state promoted by the creation of a core–shell-like structure. In this morphology, the charged interface between ZnPc **1** and DNA origami is protected from the ionic strength of the media by the hydrophilic yet neutral ethylene glycol corona.

**Figure 4:**
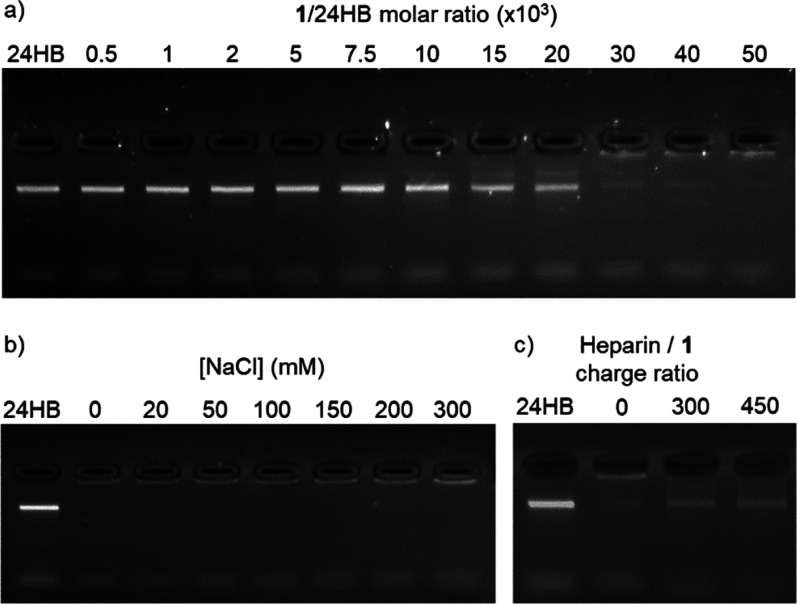
Agarose gel EMSA of (a) 24HB (2 nM) titrated with increasing molar equivalents of **1**. (b) **1**–24HB complex (100 μM and 2 nM, respectively) in the presence of increasing NaCl concentrations. (c) **1**–24HB complex (70 μM and 1.75 nM, respectively) titrated with increasing amounts of heparin.

The stoichiometric ratio required for the complex formation was determined by agarose gel electrophoretic mobility shift assay (EMSA). A constant concentration of 24HB (2 nM) was titrated with **1** to determine the maximum amount bound, i.e., the saturation ratio (Figure 4a). At increasing amounts of **1**, the intensity of the free DNA origami band decreased gradually, until an excess at a **1**/DNA origami molar ratio of ca. 40 000. A similar experiment was carried out with 60HB (Figure S3), yielding similar results.

To verify that the complexation is indeed electrostatically driven, different NaCl concentrations were added before mixing **1** and 24HB. A **1**/DNA origami molar ratio of 40 000 was selected for this experiment, to ensure full coverage of the DNA origami. The resulting EMSA indicated that **1** binds to the DNA origami up to 50 mM NaCl (Figure S4b,d). Increasing the NaCl concentration above 100 mM hindered the complexation, as revealed by the reappearance of an intense DNA origami band, thus confirming the electrostatically driven assembly. In contrast, addition of NaCl after mixing the two components showed no effect on the complex even at 300 mM (Figures 4b, S4c), which is above the biologically relevant concentrations (i.e., 150 mM). This is in good agreement with the results obtained by spectroscopy, reinforcing the hypothesis of a core–shell morphology with the nonionic triethylene glycol chains protecting the electrostatically bound interface.

The reversibility of this interaction was characterized by EMSA of **1**–24HB, titrated with negatively charged heparin (Figure 4c). This biopolymer presents a high density of negative charges (2.7 sulfate groups per disaccharide),^41,42^ establishing a competitive binding with **1**. Thus, the employed 18 kDa heparin molecules contain, on average, 90 negative charges, which can bind up to 23 tetracationic ZnPc units. At a high excess of heparin (300 negative charges of heparin for each positive charge of **1**), **1**–24HB complex formation reverses, releasing intact 24HB as observed by the identical mobility compared to intact 24HB (Figure 4c).

In this paper, the preparation of a novel Janus-type photosensitizer has been reported. The chemical design, presenting two well-defined chemical sites, renders a unique structure with very specific functionalities. On one hand, the bulky and positive moiety serves as an anchoring group for the recognition of negatively charged biomolecules. At the same time, it hinders the aggregation of ZnPc in a wide range of aqueous media, including milli-Q water, high NaCl concentrations, or 1x PBS buffer. On the other hand, the water-soluble yet neutral TEG chains provide aqueous solubility and aid to maintain electrostatic interactions even in high ionic strength, important for the future development of electrostatically directed photoactive biohybrids and their stability in biologically relevant media. When compared to nonsubstituted ZnPc, **1** shows matching photophysical properties such as fluorescence and ^1^O_2_ quantum yield.

ZnPc **1** shows efficient and reversible electrostatic binding to DNA origami structures, which is a prerequisite for the preparation of origami complexes relevant in photodynamic therapy. The resulting biohybrids show remarkable resilience toward disassembly triggered by the ionic strength of the media. In summary, in this work we took the first steps toward design and synthesis of Janus-type Pc materials with resilient optical properties and targeted binding toward biomolecules. Despite the complex stability in different aqueous media, the binding occurs only in a narrow set of conditions, limiting their application in biological media. Thus, refined structures are desirable to, first, increase the binding affinity to biomolecules, and second, to explore interactions other than electrostatics to achieve better selectivity.

## Experimental Procedures

### Materials

Chemicals and equipment are detailed in Supporting Information.

### Synthetic Protocols

#### DNA Origami

24HB and 60HB were obtained, purified, and characterized as described in Supporting Information.

#### Phthalonitrile **A**

It was synthesized adapting previously reported methods.^38^

#### Phthalonitrile **B**

Into a two-necked round-bottom flask, equipped with a stirrer, were placed 4,5-dibromo-1,2-di(tri(ethylenelycol) monomethyl ether (3 g, 5.35 mmol) and copper cyanide (3.83 g, 0.042 mol). Dry DMF (80 mL) was added, and the reaction was stirred 24 h at 150 °C. After that, 200 mL of NH_4_OH was added, and the solution was stirred for 24 h. Then the solution was extracted with ether, washed three times with water, dried over MgSO_4_, and filtered, and the solvent was vacuum evaporated, to afford the pure compound as a colorless oil. Yield: 0.48 g, 20%. ^1^H NMR (300 MHz, DMSO-*d*_6_): δ (ppm) = 7.73 (s, 2H; H_ar_), 4.3–4.2 (m, 4H; H_ar_OCH_2_), 3.8–3.7 (m, 4H; OCH_2_C*H*_2_O), 3.60 (m, 4H; OC_2_*H*_4_OC_2_*H*_4_OCH_3_), 3.5 (m, 8H; OC_2_*H*_4_OC_2_*H*_4_OCH_3_), 3.4 (m, 4H; OC_2_*H*_4_OC_2_*H*_4_OCH_3_), 3.22 (s, 6H; OCH_3_). ^13^C NMR (75 MHz, DMSO-*d*_6_): δ (ppm) = 151.79, 117.34, 116.25, 107.38, 71.25, 70.02, 69.80, 69.57, 69.06, 68.51, 58.02. FT-IR (film): *v* (cm^–1^) = 2870, 2227, 1740, 1587, 1515. MS (FB+): Calcd for C_22_H_32_N_2_O_8_Na [M + Na]^+^: *m*/*z*: 475.2, found 475.2.

#### ZnPc **2**

Compound **B** (0.2 g, 0.44 mmol), compound **A** (0.041 g, 0.06 mmol), and Zn(OAc)_2_ (0.022 g, 0.12 mmol) were placed into a sealed tube, equipped with a stirrer, and DMAE (2 mL) and DBU (0.1 mL) were added. The reaction was stirred 36 h at 140 °C. After that, the solvent was evaporated, and the crude product was triturated with ether and filtered. Then ZnPc **2** was purified on a chromatographic column of silica gel, using CHCl_3_/MeOH (10:0.5) as eluent with 1% of pyridine, followed size-exclusion chromatography with Biobeads using CHCl_3_ as eluent, to afford a dark green sticky solid. Yield: 0.015 g, 12%. ^1^H NMR (500 MHz, CDCl_3_): δ (ppm) = 8.90–8.86 (bs, 4H; H2′′), 8.79–8.68 (bs, 4H; H6′′), 8.07 (s, 2H; H3, H6), 8.02 (d, *J* = 4 Hz, 4H; H3′, H5′), 7.99 (s, 2H; H4′), 7.51 (s, 6H; H_ar_), 6.74 (dd, *J* = 5 Hz, *J* = 8 Hz, 4H; H5′′), 4.7–4.5 (m, 12H; H_ar_OC*H*_2_), 4.1–4.0 (m, 12H; OCH_2_C*H*_2_O), 3.9–3.8 (m, 12H; OC_2_*H*_4_OC_2_*H*_4_OCH_3_), 3.7 (m, 12H; OC_2_*H*_4_OC_2_*H*_4_OCH_3_), 3.6 (m, 12H; OC_2_*H*_4_OC_2_*H*_4_OCH_3_), 3.5 (m, 12H; OC_2_*H*_4_OC_2_*H*_4_OCH_3_), 3.3–3.2 (m, 18H; OC*H*_3_). FT-IR (film): *v* (cm^–1^) = 2870, 1720, 1602, 1489. MS (HR-MALDI TOF): Calcd for C_106_H_120_N_12_O_26_Zn [M]^+^: *m*/*z*: 2040.7723, found 2040.7746. UV–vis (DMSO): λ_max_ (nm) (log ε) = 679 (4.66), 614 (3.84), 365 (4.27).

#### ZnPc **1**

ZnPc **2** (0.012 g, 5.8 μmol) was placed into a sealed tube, equipped with a stirrer, and distilled DMF (2 mL) was added under argon atmosphere. Then CH_3_I was added carefully, and the reaction was stirred 12 h at 120 °C. After that, the solvent was evaporated, and the crude product was triturated with ether, filtered, and washed with MeOH, to afford a green sticky solid. Yield: 0.013 g, 87%. ^1^H NMR (500 MHz, DMSO-*d*_6_): δ (ppm) = 9.33 (s, 4H; H2′′), 8.97 (s, 4H; H6′′), 8.83 (s, 2H; H3, H6), 8.60 (s, 2H; H4′), 8.49 (d, J = 6 Hz, 4H; H3′, H5′), 8.28 (d, J = 8 Hz, 4H; H4′′), 7.78 (m, 6H; H_ar_), 7.69 (t, J = 8 Hz, 4H; H5′′), 4.7 (m, 12H; H_ar_OC*H*_2_), 4.18 (s, 12H; CH_3_), 4.07 (s, 12H; OCH_2_C*H*_2_O), 3.8 (m, 12H; OC_2_*H*_4_OC_2_*H*_4_OCH_3_), 3.7–3.6 (m, 12H; OC_2_*H*_4_OC_2_*H*_4_OCH_3_), 3.6 (m, 12H; OC_2_*H*_4_OC_2_*H*_4_OCH_3_), 3.5–3.4 (m, 12H; OC_2_*H*_4_OC_2_*H*_4_OCH_3_), 3.2 (m, 18H; OCH_3_). FT-IR (film): *v* (cm^–1^) = 3435, 3009, 2926, 2778, 2562, 2504, 2459, 2424, 1715, 1632, 1604. MS (HR-MALDI TOF): Calcd for C_110_H_132_N_12_O_26_Zn [M]^4+^: *m*/*z*: 525.2161, found 525.2171; calcd for C_110_H_132_N_12_O_26_ZnI [M + 3I]^3+^: *m*/*z*: 742.5898, found 742.5909; Calcd for C_110_H_132_N_12_O_26_ZnI_2_ [M + 2I]^2+^: *m*/*z*: 1177.3373, found 1177.3394. UV–vis (DMSO): λ_max_ (nm) (log ε) = 685 (4.79), 614 (4.05), 365 (4.80).

### Methods

Fluorescence quantum yield was measured by adapting the method of Williams,^43^ using the following equation:
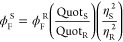
where *Φ*_F_ is the fluorescence quantum yield, S is the sample, and R is the reference. Quot is the quotient of the integrated fluorescence intensity and the absorption at the excitation wavelength of the sample, and η is the refractive index of the solvent. Φ_F_ of ZnPc in DMSO is 0.18. ZnPc **1** and ZnPc were irradiated at 665 nm.

#### Singlet Oxygen Quantum Yield

φ_Δ_ has been calculated using a relative method, where 1,3-diphenylisobenzofuran (DPBF) decomposes by the presence of ^1^O_2_. Nonsubstituted ZnPc was used as reference, that presents a φ_Δ(DMSO)_ = 0.67. Three milliliters of a stock solution of DPBF (OD ca. 1) in DMSO was placed into a 10 × 10 mm quartz optical cell, and it was bubbled with ^3^O_2_ for 1 min. A solution of ZnPc **1** with 0.1 au of absorbance at the Q-band was then added. Using a halogen lamp of 300 W, the mixture was irradiated under stirring, for defined intervals of time, for a decrease of the DPBF absorbance of 3–4% at 414 nm. A filter was used to remove light under 530 nm (Newport filter FSQ-OG530), and neutral density filters to remove light (FBS-ND03 or FB-ND10) were used when it was necessary. Experiments were performed in triplicate, expressing the final results as an average. φ_Δ_ of ZnPc **1** was calculated using the following equation:
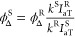
where *φ*_*Δ*_ is the fluorescence quantum yield, S is the sample, R is the reference, *k* is the slope of a plot of ln(*A*_0_/*A*_*t*_) versus irradiation time, and *A*_0_ and *A*_*t*_ the absorbance of DPBF before and after irradiation time (*t*) respectively. *I*_aT_ is the total amount of light that the dye absorbs and is calculated as a sum of intensities of the absorbed light *I*_a_ at wavelengths from the filter cutoff to 800 nm (step 0.5 nm). *I*_a_ at one determined wavelength can be determined by the Lambert–Beer law:
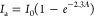
where *A* is the absorbance of the photosensitizer at the determined wavelength, and *I*_0_ the transmittance of the filter at the same wavelength.

### Biohybrid Formation

#### Electrophoretic Mobility Assay

The ZnPc–DNA origami complexes were prepared by mixing the DNA origami solution (final concentration of 2.0 nM) with increasing amounts of ZnPc, rendering ZnPc/DNA origami ratios from 500 to 50 000. The final DMSO concentration was adjusted to 10% for all samples. The mixtures were incubated at room temperature for 45 min. The total sample volume was 20 μL, and 4 μL of gel loading dye (6×) was added before loading 22 μL of the total sample into the gel pockets. The gel was visualized using a BioRad ChemiDoc MP Imaging system, and the gel was excited at 532 nm (Alexa 546) and 633 nm (Alexa 647) (Figure S3).

#### UV–Vis Spectroscopy

The change in the absorbance spectra of ZnPc in the presence of DNA origami structures and NaCl was studied with UV–vis spectroscopy. The samples were initially prepared using 100 μL PCR tubes and then transferred to a clear flat-bottom 96-well plate. The ZnPc concentration was kept constant at 7.5 μM, while the DNA origami concentration varied between the samples to obtain ZnPc/DNA origami ratios of 1000, 2000, 5000, 10 000, 20 000, 30 000, 40 000, and 50 000 which correspond to final DNA origami concentrations of 7.5, 3.75, 1.5, 0.75, 0.38, 0.25, 0.19, and 0.15 nM, respectively. Samples without DNA origami structures were also prepared as reference. First, the DNA origami solution was diluted in their respective 1× FOB to obtain the desired DNA origami concentration, after which ZnPc was added. The final DMSO concentration was adjusted to 10% for all samples. The samples were incubated for 45 min before NaCl was added in final concentrations of 50, 100, 200, and 500 mM.

#### Reversible ZnPc–DNA Origami Binding

To recover the DNA origami and dissociate the binding process, heparin sodium salt was used. The **1**–24HB complexes were prepared by mixing the 24HB solution (final concentration of 1.75 nM) with **1** (final concentration of 70 μM) to obtain a ZnPc **1**/24HB ratio of 40 000. The mixture was incubated at room temperature for 45 min. After the incubation, different amounts of heparin sodium salt were added to obtain final concentrations of 933 and 1400 μM, which corresponds to approximately 300- and 450-times higher charge concentration than that of ZnPc **1**. After addition of heparin, loading dye (6 μL) was added before loading the sample into the gel. The ZnPc–DNA origami complexes were prepared by mixing the DNA origami solution (final concentration of 2.0 nM) and the required amount of NaCl to prepare samples with final NaCl concentrations of 20, 50, 100, 150, 200, and 300 mM. The final DMSO concentration was adjusted to 10% for all samples. After that, ZnPc (final concentration of 100 μM) was added at a ZnPc/DNA origami ratio of 50 000 before the mixture was incubated at room temperature for 90 min (Figure S4a,b). In another gel, only the order of mixing was changed. NaCl was added after the formation of the complex, i.e., after the initial incubation of ZnPc–DNA origami complex for 45 min, different amounts of NaCl (same as above) were added. The sample was then incubated for an additional 45 min. The total sample volume was 20 μL, and 4 μL of gel loading dye (6×) was added before loading 22 μL of the total sample into the gel pockets (Figure S4c,d).

## Supporting Information Available

Chemicals, equipment, DNA origami folding protocols, biohybrid formation and dissociation, EMSA gels, and absorption spectra (PDF)

## Abbreviations

Pc: phthalocyanine
^1^O_2_: singlet oxygen
HB: helix bundle
DPBF: diphenylbenzofurane
φ_F_: fluorescence quantum yield
φ_Δ_: singlet oxygen quantum yield
