# Supplementary material for: A Janus-Type Phthalocyanine for the Assembly of Photoactive DNA Origami Coatings
Source: Bioconjug Chem. 2021 May 24;32(6):1123–9. doi: 10.1021/acs.bioconjchem.1c00176 (PMC8382221; doi:10.1021/acs.bioconjchem.1c00176)
Supplement: Supplementary file 1 — bc1c00176_si_001.pdf [file bc1c00176_si_001.pdf]

Supporting Information

A Janus-Type Phthalocyanine for the Assembly of Photoactive DNA Origami Coatings

Asma Rahali, [‖ ‡ †] Ahmed Shaukat, [ʃ †] Verónica Almeida-Marrero, [‖] Bassem Jamoussi, [⊥] Andrés de la Escosura, [‖ §] Tomás Torres,* [‖ § ∓] Mauri A. Kostiainen,* [ʃ] Eduardo Anaya-Plaza*[ʃ]

[‖] *Department of Organic Chemistry, Universidad Autónoma de Madrid (UAM), Calle Francisco Tomás y Valiente 7, 28049 Madrid, Spain.*

[‡] *Didactic Research Laboratory of Experimental Sciences and Supramolecular Chemistry (UR17ES01), University of Carthage, Faculty of Sciences Bizerte. Zarzouna, 7021 Bizerte, Tunis.*

[ʃ] *Department of Bioproducts and Biosystems, Aalto University, Kemistintie 1, 02150 Espoo, Finland*

[⊥] *Department of Environmental Sciences, Faculty of Meteorology, Environment and Arid Land Agriculture King Abdulaziz University, 21589 Jeddah, Saudi Arabia*

[§] *Institute for Advanced Research in Chemical Sciences (IAdChem). Universidad Autónoma de Madrid (UAM). Campus de Cantoblanco, 28049 Madrid, Spain.*

[∓] *IMDEA-Nanociencia. Campus de Cantoblanco, 28049 Madrid, Spain.*

1. Contents

2. Experimental section S2
  2.1 Chemicals S2
  2.2 Equipment S2
  2.3 Methods S2
    2.3.1 Fluorescence quantum yield S2
3. DNA origami S3
  3.1 Synthesis of DNA origami S3
  3.2 Purification of DNA origami S3
  3.3 Concentration of DNA origami S3
  3.4 Electrophoretic mobility shift assay (EMSA) S3
  3.5 Spin filtering S4
  3.6 Transmission Electron Microscopy (TEM) S4
4. Formation of ZnPc-DNA origami complexes S4
  4.1 EMSA S4
  4.2 Reversible ZnPc- DNA origami binding S4
  4.3 UV-Vis spectroscopy S5
5. Characterization S7

2. Experimental section

2.1 Chemicals

All chemical reagents were purchased from commercial sources (Sigma Aldrich, Alfa Aesar, Acros Organic, TCI) and used without further purification. All reactions were performed in standard glassware under argon atmosphere. Microwave reactions were carried out in a CEM Discovery Apparatus, in closed glass vials (Biotage 2 - 5ml) under argon atmosphere. Anhydrous solvents were dried with molecular sieves. Reactions were monitored using thin-layer chromatography (TLC) plates pre-coated with silica gel 60-F254 (Merck). Column chromatography was performed on Merck silica gel 60, 40-63 μm (230-400 mesh), and Biorad Biobeads SX-1 (200-400 mesh) were used as stationary phase for Size Exclusion Chromatography (SEC). The single-stranded scaffolds (p7249 and extended p7560) were obtained from Tilibit Nanosystems and the short single-stranded staple strands were bought from Integrated DNA Technologies. The 50× TAE Buffer (2 M tris(hydroxymethyl)aminomethane (Tris), 50 mM ethylenediaminetetraacetic acid (EDTA) and 1 M acetic acid for adjusting the pH to 8.0) was purchased from Thermo Fisher Scientific. Agarose was purchased from Bioline, 6× Gel Loading Dye Blue from New England Biolabs, sodium chloride from VWR Chemicals, magnesium chloride hexahydrate, dimethyl sulfoxide and ethidium bromide from Sigma-Aldrich. The water used in all the experiment was Milli-Q grade.

2.2 Equipment

^{1}H and ^{13}C-NMR spectra were performed using Bruker DRX 500, Bruker Avance and Bruker Avance II spectrometers (^{1}H: 300 MHz and 500 MHz ^{13}C: 75 MHz and 125 MHz, respectively). The internal references of all spectra were made using the residual solvent (^{1}H: δ = 7.26 for $CDCl_3$, δ = 2.49 for $DMSO_{d6}$) or solvent resonances (^{13}C: δ = 77.0 for $CDCl_3$) relative to $SiMe_4$, respectively (*s*: singlet, *bd*: broad singlet, *d*: doublet, *dd*: double doublet, *t*: triplet, *m*: multiplet, H_{ar}: aromatic protons). Deuterated solvents that were employed are indicated in parentheses. Testing temperature of 295 K was employed, unless otherwise stated.

Mass experiments were done at the Servicio Interdepartamental de Investigación (SIdI) of the Universidad Autónoma de Madrid, by Matrix-assisted laser desorption/ionization time of flight (MALDI-TOF), that was recorded using a Bruker Ultrareflex III spectrometer; Electrospray Ionization Source (ESI) in positive or negative mode, that was performed using an ultra-high-resolution QTOF instrument (MAXIS II, Bruker), and Fast Atom Bombardment (FAB) was recorded using a Waters – VG AutoSpec spectrometer. UV-Vis absorbance and fluorescence were recorded using JASCO V-660 and JASCO FP-8600 spectrophotometers, respectively. Infrared spectra (IR) were recorded on a Cary630 FTIR spectrophotometer from Agilent Technologies, using solid sample (Diamond ATR); spectra intensity was corrected by ATR algorism and the subsequent baseline correction.

The DNA origami structures were folded using G-storm G1 Thermal Cycler. For purification purpose, Eppendorf 5424R microcentrifuge was utilized. The absorbance for determining the concentration of DNA origami was measured using a BioTek Eon Microplate spectrophotometer and a Take3™ micro-volume plate. The Electrophoretic mobility shift assay were done using BioRad horizontal wide Mini-Sub GT electrophoresis system and a BioRad PowerPac™ Basic power supply. The gels were visualized under UV light using a BioRad Gel Doc™ XR+ Documentation system. The transmission electron microscopy images of the biohybrids were taken using an FEI Tecnai 12 Bio-Twin instrument operated at an acceleration voltage of 120 kV and were processed using Gatan Digital Micrograph software. For UV-Vis spectroscopy, Cytation 3 plate reader (BioTek) was used.

2.3 Methods

2.3.1 Fluorescence quantum yield

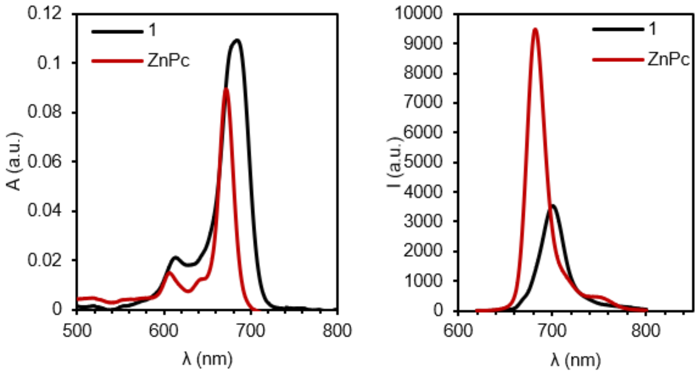

Figure S1. Absorption and fluorescence spectra for fluorescence quantum yield calculations. λ_{exc} = 665 nm.

3. DNA origami

3.1 Synthesis of DNA origami

The 24-helix bundle (24HB) and 60-helix bundle (60HB) synthesis have been adapted from Ijäs *et al.*[1] and Linko *et al.*[2] respectively. The scaffold used for folding 24HB and 60HB is 7560 and 7249 nucleotides respectively. For 24HB, there are 202 oligonucleotides staple strands with 8× poly-T overhang whereas, the 60HB design is composed of 141 oligonucleotides staple strands. The exact sequence can be extracted from the above-mentioned references. The folding buffer (FOB) utilized for 24HB was in 1x TAE, 17.5 mM $MgCl_2$ and while, 60HB was folded in buffer containing 1x TAE, 20 mM $MgCl_2$ and 5 mM NaCl. The volume and final concentration have been tabulated in Table 1. Same thermal annealing was used for both the DNA origami which was initiated after mixing all the ingredients in PCR tubes. The protocol was as follows:

- Heating the mixture to 65 °C,
- followed by, cooling the mixture from 65 °C to 60 °C over 90 minutes
- and then cooling slowly from 60 °C to 39 °C over 60 hours.

After the folding procedure, the DNA origami structures were kept at 12 °C until the program was manually stopped.

Table S1. The amounts and the concentrations (initial and final) of the components used for the folding of the 24HB and 60HB structure.

	**24HB**			**60HB**		
Compound	Volume	$C_{Initial}$	C_{Final}	Volume	$C_{Initial}$	C_{Final}
Scaffold	20 µL	100 nM	20 nM	20 µL	100 nM	20 nM
Staple Strands	40 µL	500 nM	200 nM	40 µL	500 nM	200 nM
TAE Buffer	10 µL	10x	1x	10 µL	10x	1x
$MgCl_2$	30 µL	58.3 mM	17.5 mM	20 µL	100 mM	20 mM
NaCl	-	-	-	10 µL	50 mM	5 mM
Total	**100 µL**			**100 µL**		

3.2 Purification of DNA origami

The folded DNA origami structures were purified by a poly(ethylene glycol) (PEG) purification method. The method was adapted from Stahl *et al.*[3] Two folding reactions (i.e. 200 µL) were then mixed with 600 µL of 1× FOB to obtain a starting volume of 800 µL. The solution was mixed thoroughly with 800 µL of PEG precipitation buffer (15 % PEG 8000 (w/v), 1× TAE and 505 mM NaCl), after which the mixture was centrifuged at 14000 rcf for 30 minutes at room. After the centrifugation, the supernatant was removed, and the DNA origami pellet was redissolved in 1× FOB (same as described above for 24HB and 60HB). Depending on the required DNA concentration, the pellet was redissolved in a different amount of 1× FOB to get final concentrations between 30 nM and 50 nM. The DNA origami solution was incubated at room temperature overnight to ensure complete dissolution of the pellet before refrigerated.

3.3 Concentration of DNA origami

The concentration was measured via UV/Vis spectroscopy and determined using Beer-Lambert law.

$A_{260} = \varepsilon\, C_{DNA}\, l$

where A_{260} is the absorbance at 260 nm, ε is the molar extinction coefficient (for the 24HB and 60HB structure 1.05×10^8 M^{-1} $cm^{-1}$and 0.9×10^8 M^{-1} cm^{-1} respectively) and l is the path length through the solution in centimeters (0.05 cm). A sample size of 2 µL was used for the measurements.

3.4 Electrophoretic mobility shift assay (EMSA)

Agarose gel electrophoresis was used to analyze the folded 24HB and 60HB structures, and to verify the removal of excess amounts of staple strands after the PEG purification. A 2% (w/v) agarose gel was prepared by mixing 2 g of agarose into 90 mL of 1× TAE buffer and heated until a clear solution was obtained. After cooling it slightly, 10 mL of 110 mM $MgCl_2$ was added. The gel was stained with 80 µL of ethidium bromide (EtBr) solution (0.58 mg/mL) before being poured into the casting tray. For the DNA origami samples, 10 µL of DNA origami solution was used, whereas the scaffold reference sample was prepared by diluting 4 µL of the scaffold (type p7249 and p7560, c = 100 nM) in 6 µL of their respective 1× FOB. The samples were mixed with 2 µL of Gel Loading Dye Blue (6×) and the whole sample of 12 µL was loaded into the gel pocket. The gel was run at a constant voltage of 90 V for 40 minutes. The running buffer utilized for EMSA was 1× TAE buffer containing 11 mM $MgCl_2$ and the gel electrophoresis chamber was kept on an ice bath for the run.

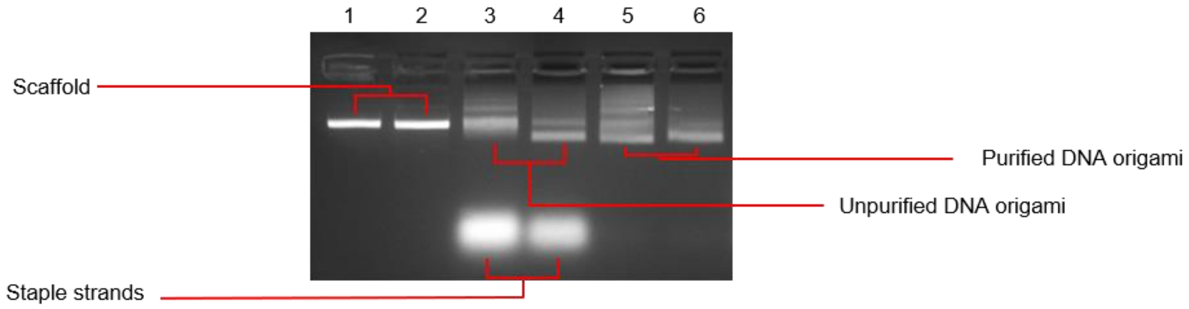

Figure S2. Qualitative analysis of 24HB and 60HB using agarose gel electrophoresis. 1) Scaffold (type p7560). 2) Scaffold (type p7249) 3) Unpurified, but folded 24HB. 4) Unpurified, but folded 60HB. 5) PEG-purified 24HB. 6) PEG-purified 60HB.

3.5 Spin filtering

The folded DNA origami were spin filtered to remove the folding buffer and redispersed in the milli-Q water. The Amicon® Ultra 0.5mL filters (100K) were first rinsed with 500 µL milli-Q water and centrifuged at 14000 rcf for 5 minutes at room temperature using an Eppendorf 5424R microcentrifuge. The follow-through is discarded and the filter is filled with 250 µL of DNA origami and 250 µL of milliQ water. The filter is then centrifuged at 6000 rcf for 10 mins. The follow-through is discarded and 480 µL milliQ water is added. The filter is then centrifuged at 6000 rcf for 10 mins. The follow-through is discarded and the filter is inverted into a new outer shell and centrifuged for 150 seconds at 1000 rcf. The filtrate is then mixed with milliQ water and the concentration is checked by the previously mentioned protocol (2.3).

3.6 Transmission Electron Microscopy (TEM)

The folded 24HB and 60HB structures were imaged using TEM. The sample was negatively stained with uranyl formate (2 % (w/v)) containing 25 mM NaOH according to the method described by Castro *et al.*[4] The sample was prepared by diluting the DNA origami solution in 1× FOB to a final concentration of 2 nM. The sample was observed using Formvar carbon-coated copper grids (400 mesh, Electron Microscopy Sciences) and were plasma cleaned (20 seconds oxygen plasma flash using a Gatan Solarus) before applying the sample. The sample volume of 3 µL was applied on the carbon-coated side of the grid for 3–3.5 minutes. After the incubation, the excess solution was drained from the edge using filter paper. First, the grid was immersed into a 5 µL droplet of uranyl formate solution, and excess liquid was directly drained off from the edge using filter paper, followed by a subsequent immersion into a 20 µL droplet of uranyl formate solution. The excess amount of stain was removed after 45 seconds from the edge of the grid using filter paper. After this process, the grid was left at room temperature for at least 30 minutes before imaging.

4. Formation of ZnPc-DNA origami complexes

4.1 EMSA

The ZnPc-DNA origami complexes were prepared by mixing the DNA origami solution (final concentration of 2.0 nM) with increasing amounts of ZnPc, rendering ZnPc/DNA origami ratios from 500 to 50 000. The final DMSO concentration was adjusted at 10% for all samples. The mixtures were incubated at room temperature for 45 minutes. The total sample volume was 20 µL and 4 µL of gel loading dye (6×) was added before loading 22 µL of the total sample into the gel pockets. The agarose gel was prepared and ran using the protocol described in Section 2.4. The gel was visualized using a BioRad ChemiDoc™ MP Imaging system and the gel was excited at 532 nm (Alexa 546) and 633 nm (Alexa 647).

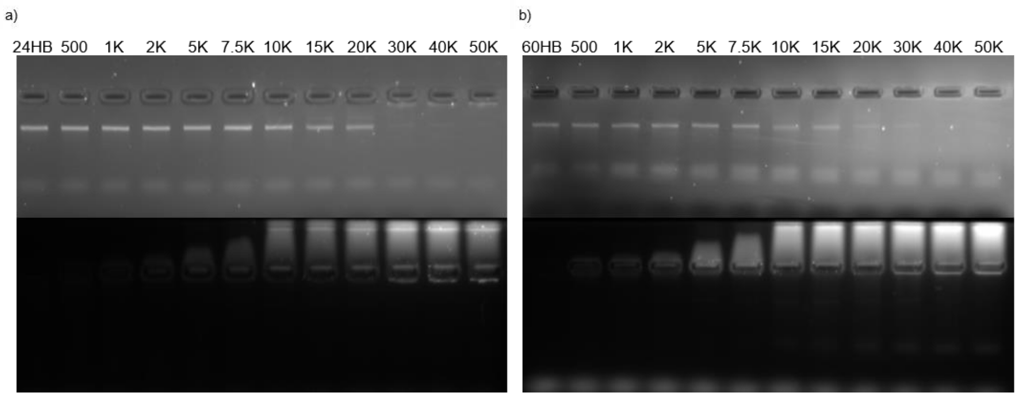

Figure S3. EMSA of a) 24HB (2 nM) b) 60HB (2nM) titrated with increasing molar equivalents of ZnPc **1**

4.2 Reversible ZnPc- DNA origami binding

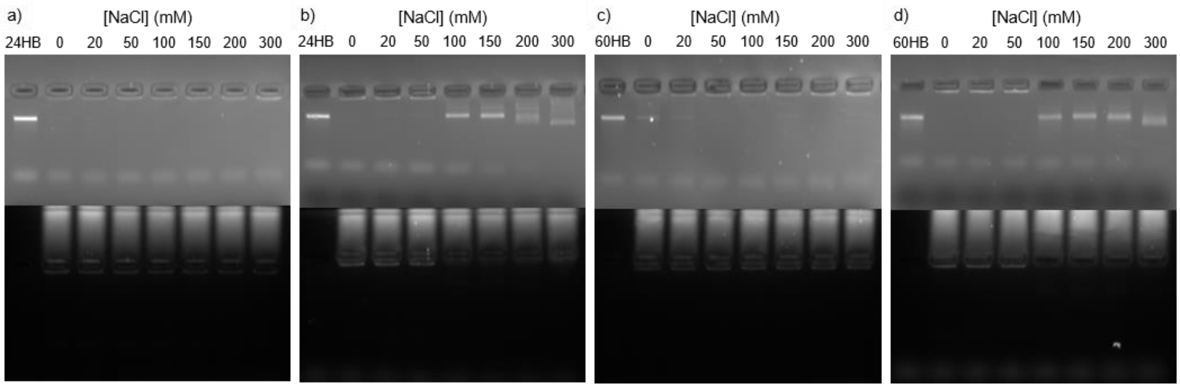

Figure S4. Agarose gel EMSA of the a–b) **1**–24HB complex and c–d) **1**–60HB complex (100 µM and 2 nM, respectively) at increasing NaCl concentrations. In a) and c) the NaCl was added after binding. In b) and d) the NaCl was added before binding.The EMSA gels show the EtBr (top) and ZnPc (bottom) emission channels recorded at 532 and 633 nm, respectively.

4.3 UV-Vis spectroscopy

The NaCl crowding effect on the ZnPc **1** was studied by absorbance, recorded on a Cytation 3 plate reader (Figure S8).

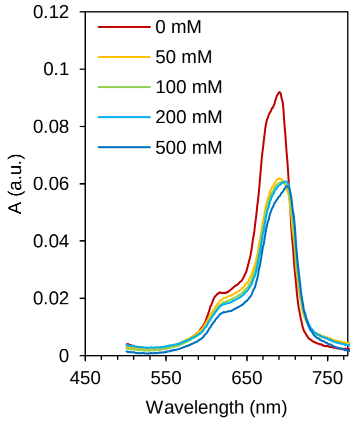

Figure S5: Absorption spectra of 7.5 µM of **1** in the presence of 0-500 mM of NaCl.

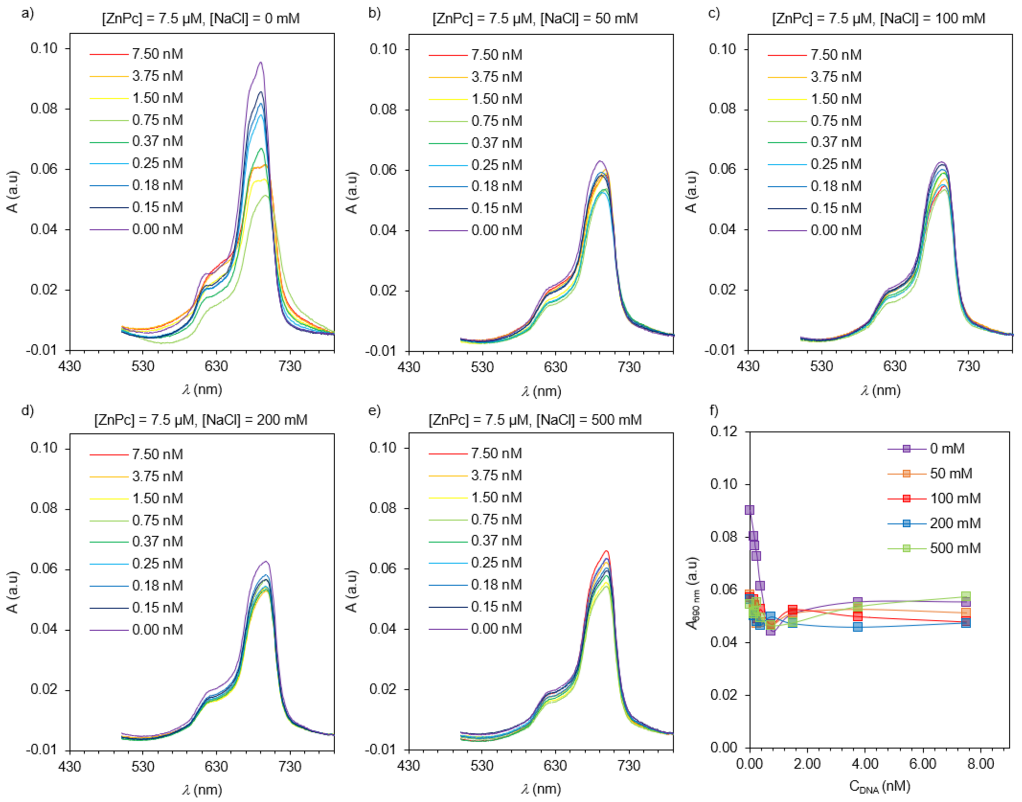

Figure S6. Absorption spectra of 7.5 µM of **1** in the presence of 0–7.50 nM of 24HB (**1**/24HB = 1000–50000) at different mM NaCl. a) 0 mM NaCl. b) 50 mM NaCl. c) 100 mM NaCl. d) 200 mM NaCl. e) 500 mM NaCl. f) Absorbance of 7.5 µM of **1** at 690 nm in the presence of different 24HB concentrations (nM) and increasing amount of NaCl (mM).

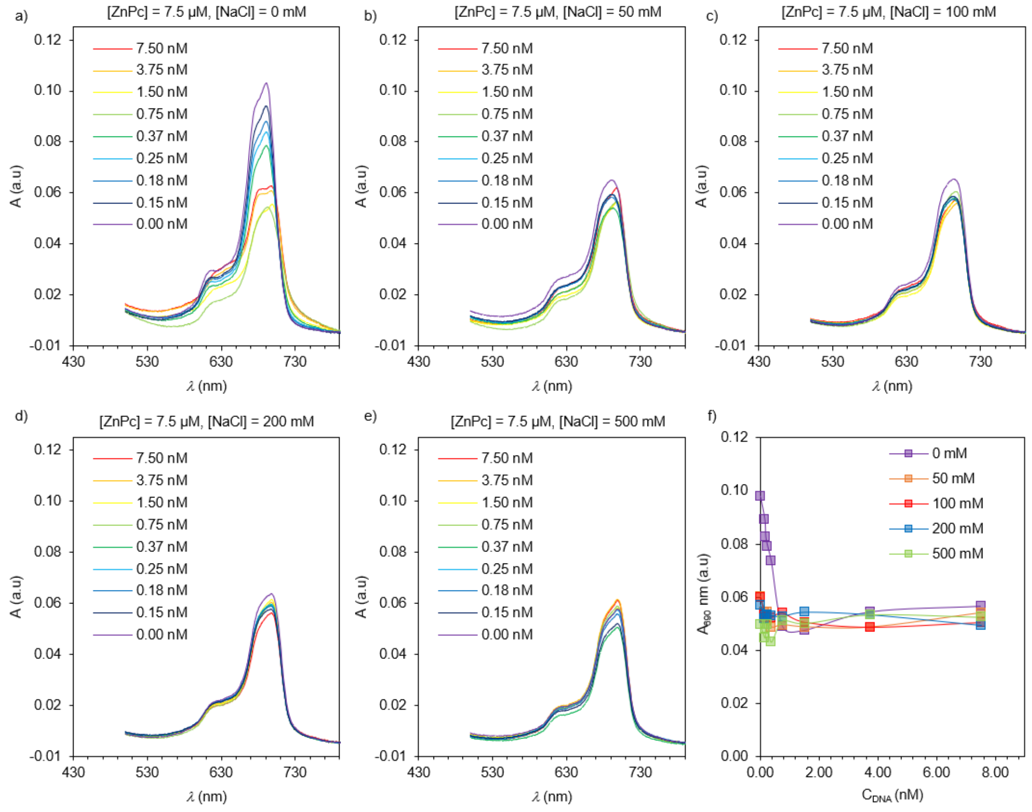

Figure S7. Absorption spectra of 7.5 µM of **1** in the presence of 0–7.50 nM of 60HB (**1**/60HB = 1000–50000) at different mM NaCl. a) 0 mM NaCl. b) 50 mM NaCl. c) 100 mM NaCl. d) 200 mM NaCl. e) 500 mM NaCl. f) Absorbance of 7.5 µM of at 690 nm in the presence of different 60HB concentrations (nM) and increasing amount of NaCl (mM).

5. Characterization

5.1 Compound B

5.1.1 ¹H-NMR

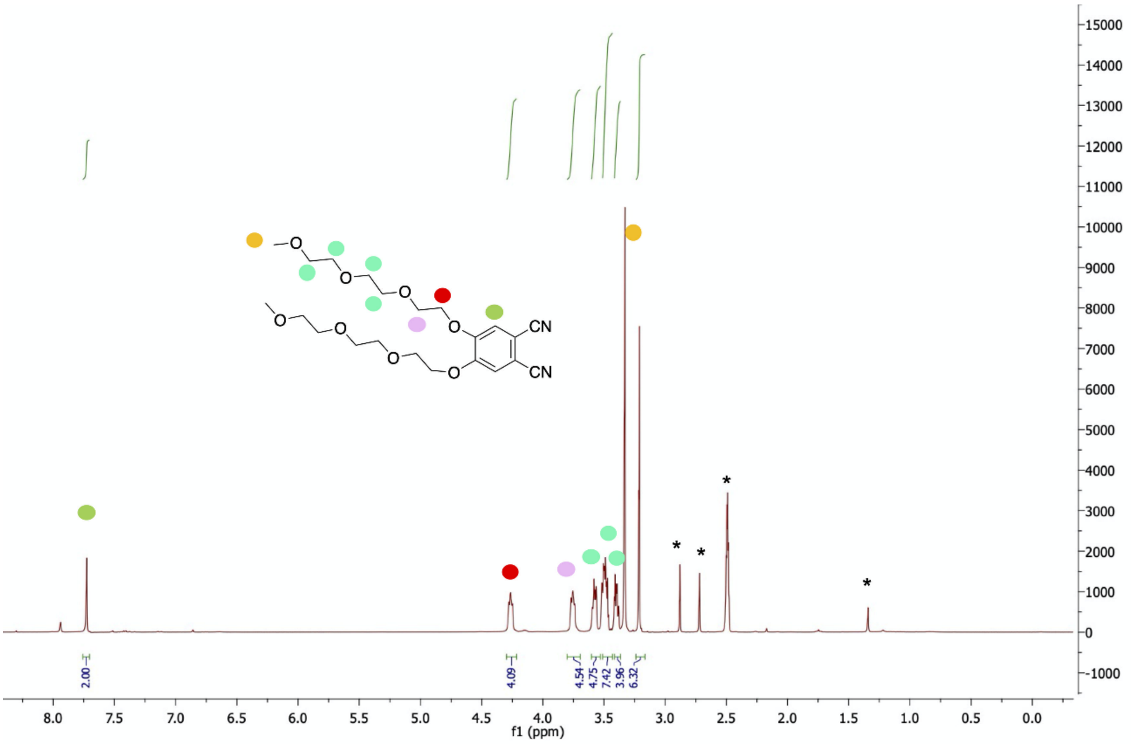

Figure S8. ¹H-NMR (300 MHz, $DMSO_{d6}$) spectra of compound B.

5.1.2 ¹³C-NMR

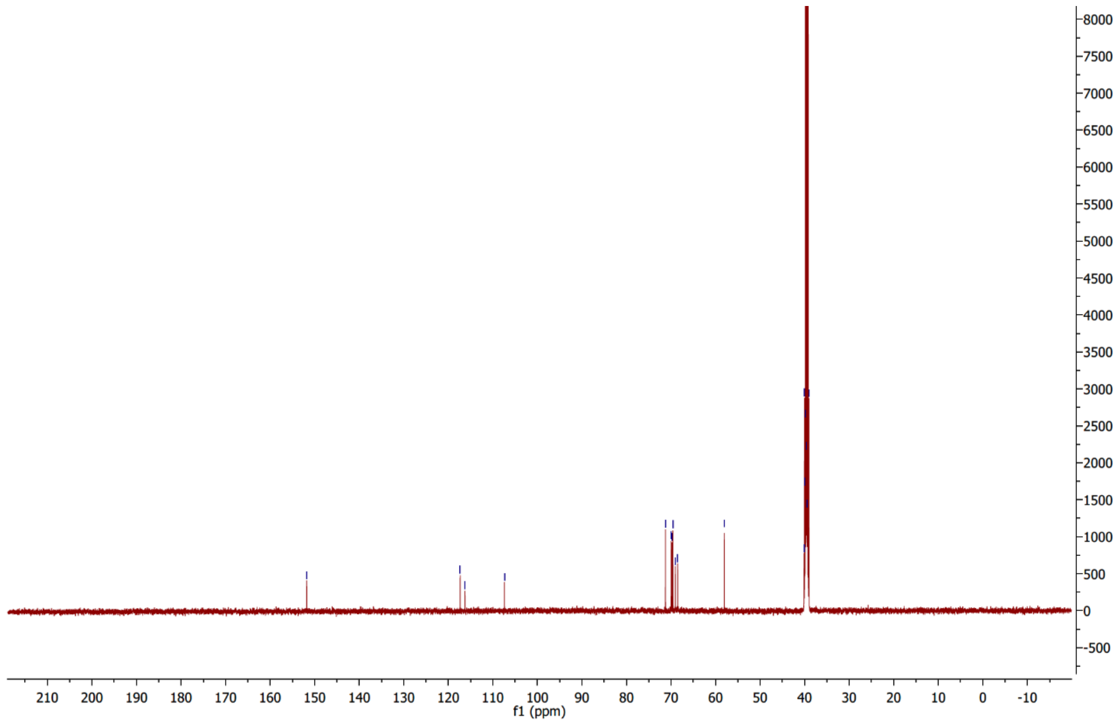

Figure S9. ¹³C-NMR (75 MHz, $DMSO_{d6}$) spectra of compound B.

5.1.3 Mass spectrum

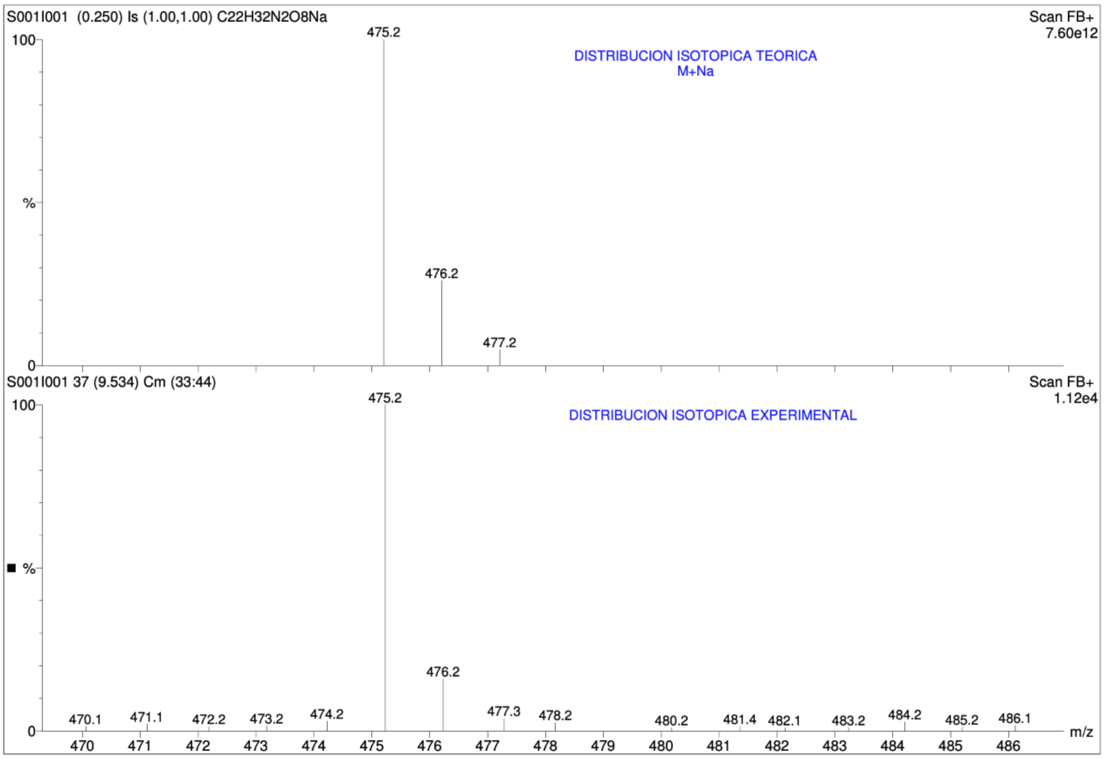

Figure S10. FAB+ mass spectrum of compound B (bottom) and calculated distribution (top).

5.2 ZnPc 2

5.2.1 ^{1}H-NMR

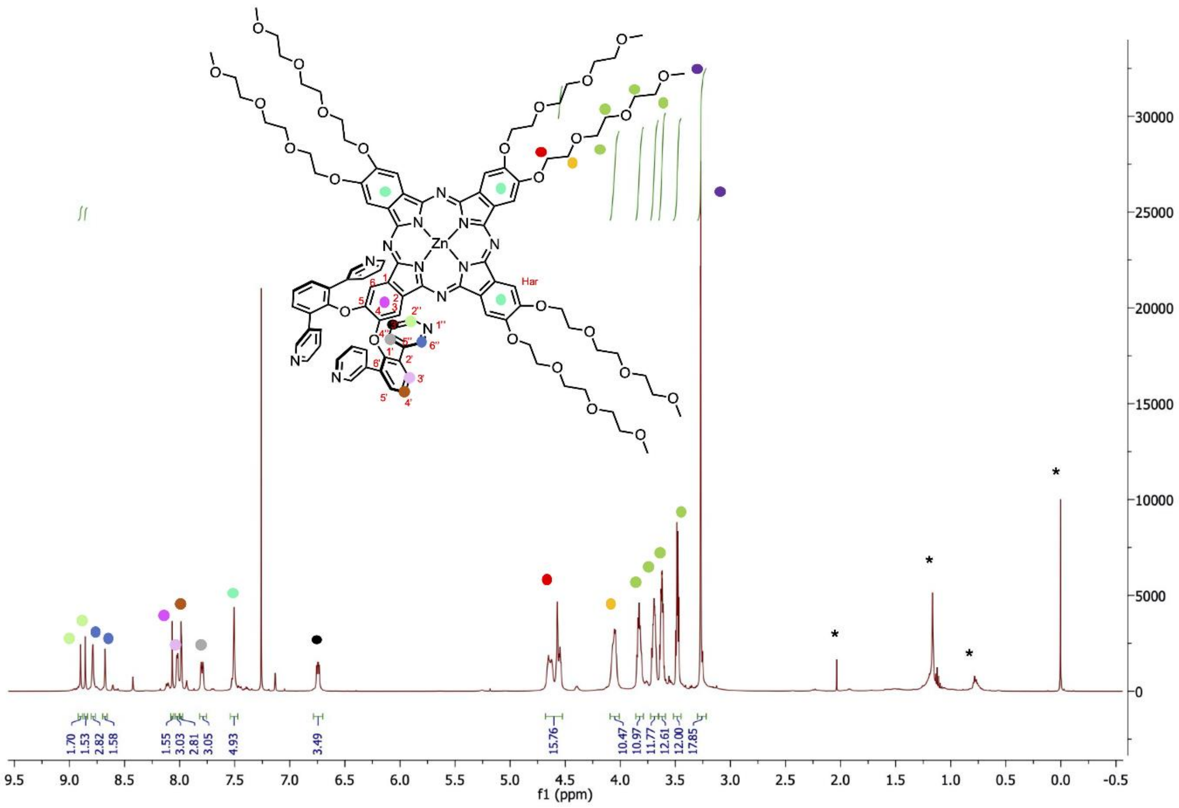

Figure S11. ^{1}H-NMR (500 MHz, $CDCl_3$) spectra of ZnPc **2**.

5.2.2 Mass spectra

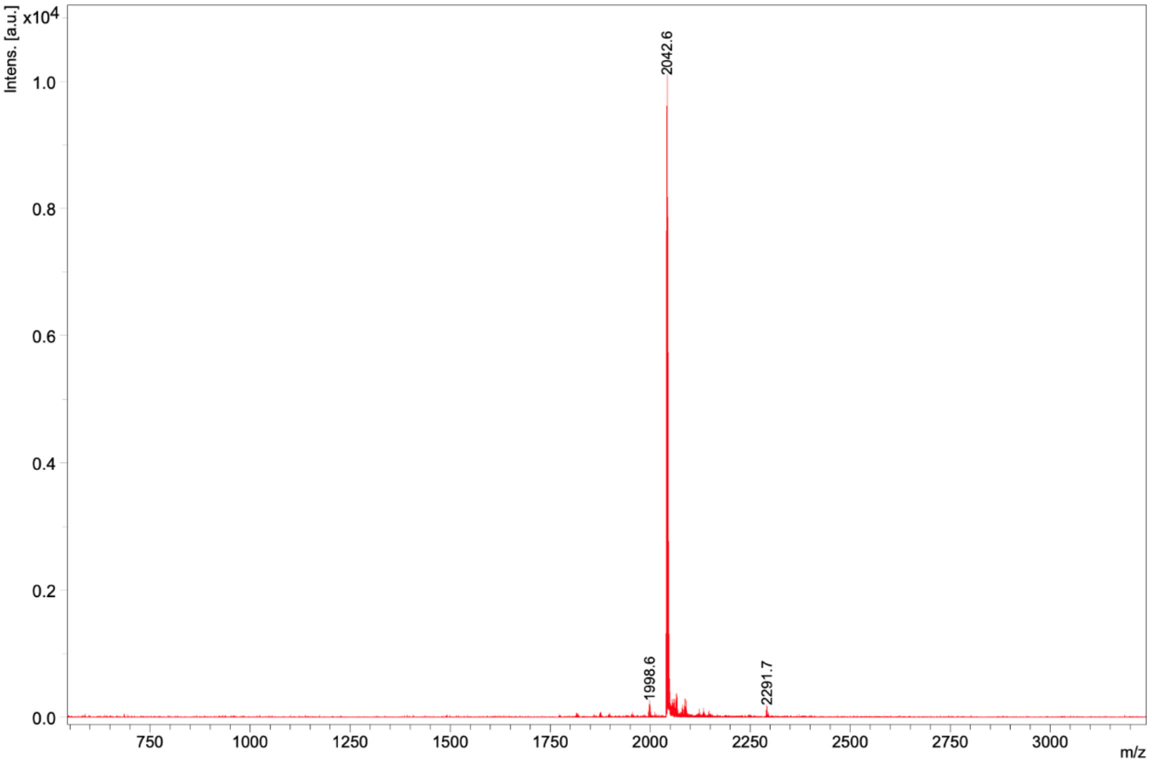

Figure S12. MALDI-TOF mass spectrum of ZnPc **2**.

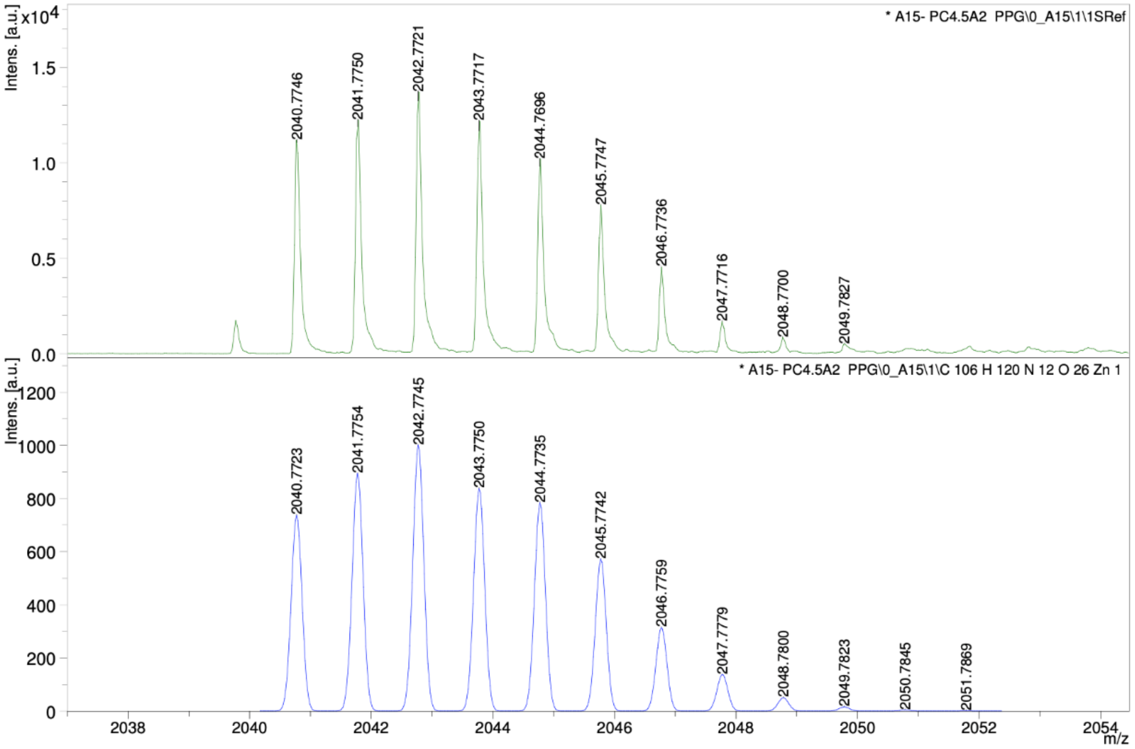

Figure S13. High-Resolution MALDI-TOF mass spectrum of ZnPc **2** (top) and calculated distribution (bottom).

5.3 ZnPc 1

5.3.1 ^{1}H-NMR

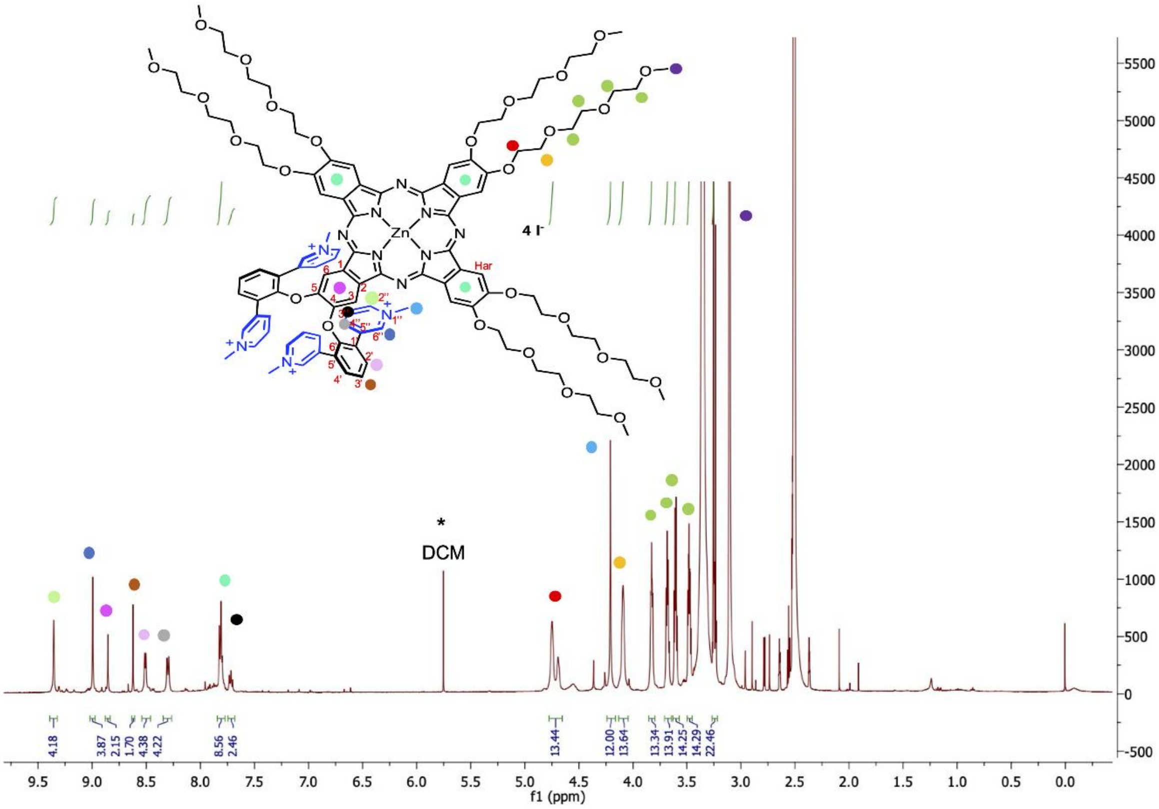

Figure S14. ^{1}H-NMR (500 MHz, $DMSO_{d6}$) spectra of ZnPc **1**.

5.3.2 Mass spectrum

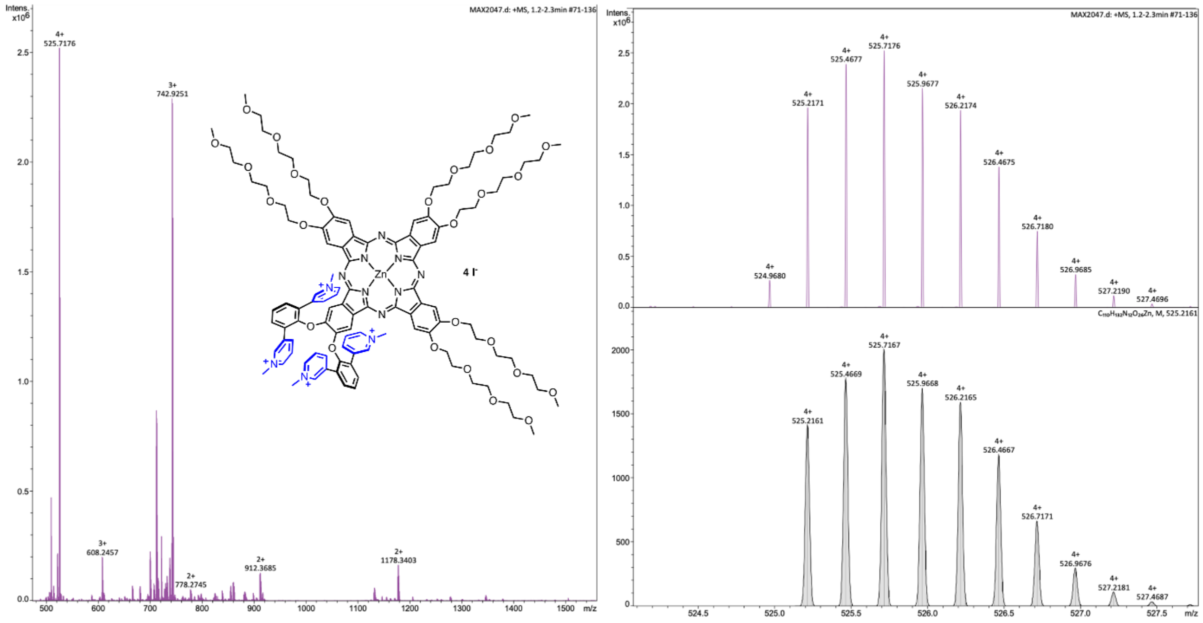

Figure S15. MALDI-TOF mass spectra of ZnPc **1** left), and high-resolution MALDI-TOF spectrum of selected peak (bottom right) and calculated distribution (top right).

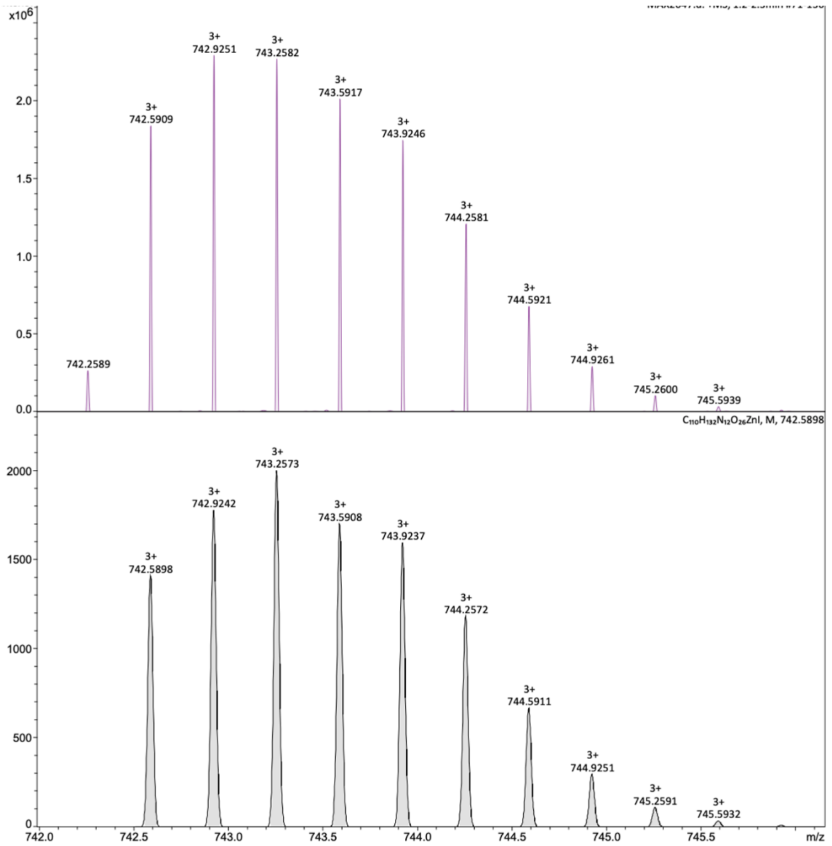

Figure S16. High-Resolution MALDI-TOF mass spectrum of selected ZnPc **1** peak (bottom) and calculated distribution (top).

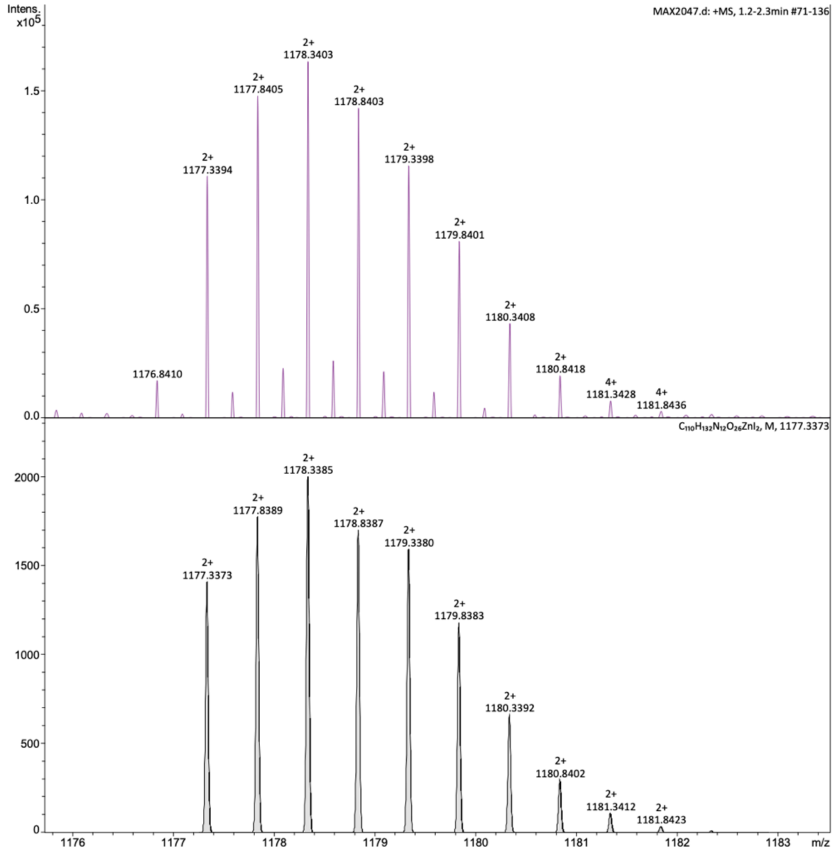

Figure S17. High-Resolution MALDI-TOF mass spectrum of selected ZnPc **1** peak (bottom) and calculated distribution (top).

6. References:

(1) Ijäs, H.; Shen, B.; Heuer-Jungemann, A.; Keller, A.; Kostiainen, M. A.; Liedl, T.; Ihalainen, J. A.; Linko, V. Unraveling the Interaction between Doxorubicin and DNA Origami Nanostructures for Customizable Chemotherapeutic Drug Release; *Nucleic Acid Res.* **2021**, *49* (6), 3048-3062.

(2) Linko, V.; Shen, B.; Tapio, K.; Toppari, J. J.; Kostiainen, M. A.; Tuukkanen, S. One-Step Large-Scale Deposition of Salt-Free DNA Origami Nanostructures. *Sci. Rep.* **2015**, *5* (1), 15634.

(3) Stahl, E.; Martin, T. G.; Praetorius, F.; Dietz, H. Facile and Scalable Preparation of Pure and Dense DNA Origami Solutions. *Angew. Chem. Int. Ed.* **2014**, *53* (47), 12735–12740.

(4) Castro, C. E.; Kilchherr, F.; Kim, D.-N.; Shiao, E. L.; Wauer, T.; Wortmann, P.; Bathe, M.; Dietz, H. A Primer to Scaffolded DNA Origami. Nat. Methods **2011**, *8* (3), 221–229.
